# Urotropin Excreted in the Bile and Pancreatic Juice

**Published:** 1908-04

**Authors:** 


### UROTROPIN EXCRETED IN THE BILE AND PANCREATIC JUICE.

---

It is with particular interest that we note the original and experimental work by Mr. Crowe, student, Johns Hopkins Medical School, along the line indicated by the above title. Mr. Crowe is an Atlanta man, and is a son of Dr. W. A. Crowe, who is a well-known and highly esteemed physician of this city.

According to the March issue of the John's Hopkins Hospital Bulletin, Mr. Crowe reported, at a recent meeting of the Hopkins Medical Society, the result of a series of experiments made to determine the fate of urotropin in the body and its efficacy as a sterilizing agent in the bile and other secretions of the body.

It was determined by experiments on dogs that after the administration of urotropin by mouth, it was excreted both in the bile and pancreatic juice.

In view of these findings, observations were made on a series of patients in the hospital who had biliary fistulæ. Bacteriological studies were made before and after giving the drug; and in every case the infecting organisms rapidly disappeared when the dose of urotropin given was 75 grains or more a day. As in the urinary bladder the organisms appear again as the dose is decreased.

The bile discharged through the fistula, when acidified and distilled, always gives the test for formaldehyde, the amount present varying with the amount of urotropin given.

In every case the patient's general condition improved, the discharged bile changed from a dirty, turbid fluid, to the golden-yellow of normal bile, and the fistulæ closed rapidly.

Urotropin was shown to be present repeatedly in the cerebrospinal fluid, even after very small doses by mouth. In one case with a badly infected cerebrospinal fistula, with sloughing and a purulent discharge, the organisms gradually disappeared after urotropin was begun, the fistula closed, and the patient made a good recovery.

Formaldehyde was shown to be present also in the pus obtained from a gonorrhœal knee-joint, but sufficient time has not as yet elapsed to report on its therapeutic effect in this case.

